# Thermal treatment of hair for the synthesis of sustainable carbon quantum dots and the applications for sensing Hg^2+^

**DOI:** 10.1038/srep35795

**Published:** 2016-10-20

**Authors:** Yongming Guo, Lianfeng Zhang, Fengpu Cao, Yumin Leng

**Affiliations:** 1 College of Chemistry and Pharmaceutical Engineering, Nanyang Normal University, Nanyang 473061, China; 2 College of Physics and Electronic Engineering, Nanyang Normal University, Nanyang 473061, China

## Abstract

A facile, simple and low-cost approach for synthesizing highly fluorescent carbon quantum dots (CQDs) from thermal treatment of sustainable hair has been developed. The resultant CQDs exhibited strong blue emission with a quantum yield of 10.75%, excellent photostability and high stability in high salt conditions. As the fluorescence of CQDs can be efficiently quenched by Hg^2+^, the CQDs can be constructed as a nanosensor for Hg^2+^ with good sensitivity and selectivity. And as low as 10 nM Hg^2+^ can be successfully detected.

Carbon quantum dots (CQDs), as an emerging type of fluorescent nanomaterial, have drawn enormous attention in recent years due to their unique properties, such as low-cost, easy synthesis, good water solubility, stable fluorescence, low toxicity and biocompatibility^1^,^2^,^3^,^4^,^5^,^6^,^7^. As an alternative to semiconductor quantum dots, CQDs have been found more promising applications in many areas including bioimaging^2^,^3^,^5^,^7^,^8^, sensors^1^,^2^,^5^,^6^, medical diagnosis^2^,^3^,^5^,^6^, photocatalysis^2^,^7^ and optoelectronics^2^,^4^,^7^. Thus, numerous methods have been developed for synthesizing a variety of CQDs during the past few years^2^,^7^, such as hydrothermal method^9^,^10^,^11^,^12^, microwave method^13^,^14^, thermal treatment method^15^,^16^, electrochemical method^17^. Among these reported approaches for CQDs synthesis, thermal treatment of biomass is a low-cost and convenient route for mass production without requirement of any solvent^18^,^19^,^20^. Human hair, a waste biomass, has been employed to synthesize CQDs via different methods^21^,^22^,^23^. Sun *et al*. have prepared CQDs with blue emission through concentrated sulfuric acid carbonization and etching of hair^21^. The method is not green and very dangerous due to the use of concentrated H_2_SO_4_ under heating condition. Chen’s group has synthesized blue emitting CQDs through one-step pyrolysis of hair at 300 °C under a nitrogen atmosphere^22^. However, high temperature and inert gas protection are required. Recently, a hydrothermal treatment of hair in water to synthesize CQDs has been developed^23^. But the yield of CQDs is only 14%. Most hair has not been fully utilized. Besides, the thermal treatment of hair at low temperature has not been used to prepare CQDs. Therefore, a facile and simple thermal treatment method for synthesizing CQDs from hair is highly desirable.

Herein, we have developed a novel and simple approach for synthesizing highly fluorescent CQDs via a one-step thermal treatment of hair. The as-prepared CQDs with a quantum yield (QY) of 10.75% showed excitation-dependent emission behavior, excellent photostability and high stability in high salt conditions. We have studied the application of CQDs as nanosensors for metal ions. The CQDs can be utilized to detect Hg^2+^ with good selectivity and sensitivity via the Hg^2+^-induced fluorescence quenching mechanism. And as low as 10 nM Hg^2+^ can be successfully detected.

## Results

### Synthesis of CQDs

**Figure 1:**
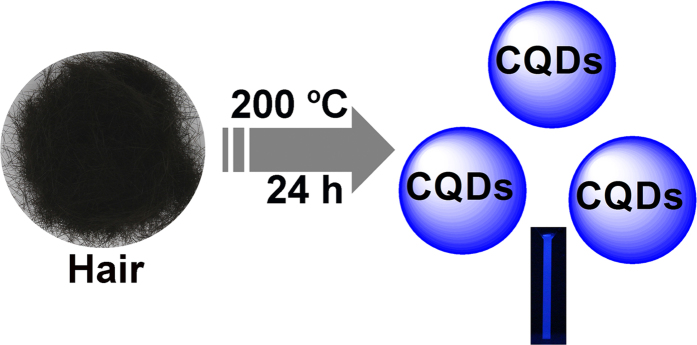
Illustration of synthesizing CQDs from hair.

Hair, composed of 99% keratin and 1% other elements^23^, can be used as carbon source for synthesizing CQDs. The CQDs were prepared via one-step thermal treatment of hair at 200 °C for about 24 hours (Fig. 1) and black solid product was obtained. After dissolution and centrifugation, the CQDs solution was obtained. The CQDs formed from the direct carbonization of hair at high temperature. At high temperature, hair first softened and further carbonized into small CQDs. As a result, the CQDs formed from hair. Moreover, the yield of the CQDs is estimated to be about 95%, which is much higher than that of the CQDs from the hydrothermal treatment of hair^23^.

### The optical properties of CQDs

**Figure 2:**
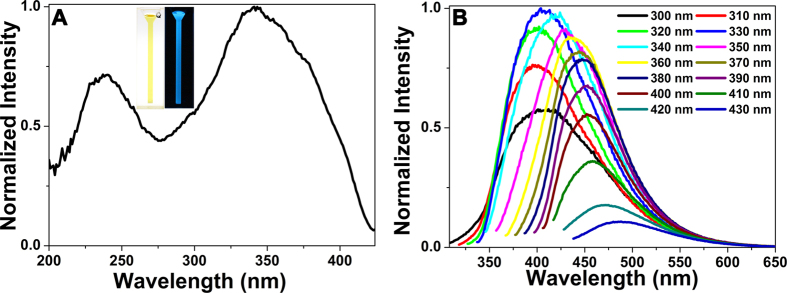
(**A**) Excitation spectrum of CQDs, inset: photographs of CQDs taken under visible light and UV light of 365 nm. (**B**) Emission spectra of CQDs at different excitation wavelengths.

The optical properties of CQDs are very important for the applications of CQDs. The color of the well-dispersed CQDs was brown in daylight, while the CQDs showed bright blue fluorescence under irradiation with 365 nm ultraviolet (UV) light (Fig. 2A). The strong blue fluorescence can be observed with the naked eye, making them promising applications in a broad range of areas. In UV-visible (UV-vis) absorption spectrum of CQDs, we observed two shoulder peaks at about 275 nm and 330 nm (Figure S1), which may be attributed to the π  → π* transition of the C = C bond and n → π* transition of the C = O bond, respectively^24^. The CQDs showed two peaks centered at about 240 nm and 330 nm in the excitation spectrum (Fig. 2A). We noticed that the CQDs exhibited an excitation-dependent emission behavior within the excitation wavelength range of 300 to 430 nm, indicating that the emission of CQDs could be turned by changing the excitation wavelength. The maximum excitation wavelength and maximum emission wavelength of the CQDs were at 330 and 415 nm, respectively (Fig. 2B). Using quinine sulfate as a reference^25^, the QY of CQDs in aqueous solution at an excitation wavelength of 330 nm was calculated to be about 10.75%, which is comparable to that of graphene quantum dots^26^,^27^. However, the QY is lower than that of the CQDs from hydrothermal treatment of hair, which may be attributed to the fact that the small CQDs formed from the direct carbonization of hair can not fully interact with each other in the solid-phase synthetic procedure. As a result, the small CQDs can not further grow into uniform CQDs, resulting into non-uniform CQDs with low QY.

### The stability of CQDs

**Figure 3:**
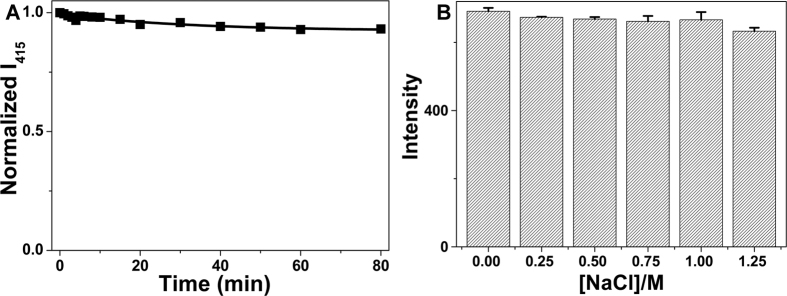
(**A**) Photostability test of CQDs under continuous irradiation of the 365 nm light. (**B**) Plot of the fluorescence intensity of CQDs at 415 nm in the presence of different concentrations of NaCl solution (performed in pH 5.0, 50 mM NaAc-HAc buffer; excitation wavelength is 330 nm).

The stability of CQDs is of great importance for the practical applications. The photostability of CQDs was demonstrated through continuous UV light (365 nm) irradiation. The fluorescence intensity showed no obvious decrease after 80 min of illumination (Fig. 3A), revealing the good photostability of CQDs. And 1.0 M NaCl did not affect the fluorescence intensity of CQDs (Fig. 3B), demonstrating the excellent stability in high-salt conditions. And the obtained CQDs showed good antioxidant activity because the fluorescence intensity of CQDs in NaClO (100 μM) solution decreased by 18% (Figure S2). These results revealed that the CQDs have a great potential in practical applications.

### Characterization of CQDs

**Figure 4:**
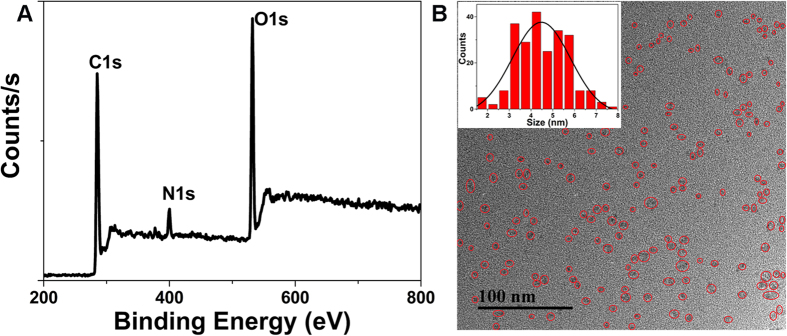
(**A**) XPS spectrum of CQDs. (**B**) TEM image of CQDs, inset: the size distribution histogram of CQDs.

We tried different techniques to elucidate the structure of CQDs. X-ray photoelectron spectroscopy (XPS) was performed to determine the surface elemental composition of CQDs. The survey XPS spectrum of CQDs showed three major peaks at about 284.6, 399.5 and 531.0 eV), corresponding to C1s, N1s and O1s, respectively (Fig. 4A). The high resolution XPS spectrum of C1s can be deconvoluted into three peaks at 284.6, 286.1 and 287.7 eV (Figure S3A), which can be ascribed to C-C/C = C, C-N/C-O and C = O bonds, respectively^28^. The N1s spectrum confirms two main bands at 399.5 eV and 401.6 eV (Figure S3B), revealing the existence of pyridinic N and pyrrolic N^29^. The O1s spectrum exhibited two peaks at 531.0 and 532.3 eV for C = O and C-O, respectively (Figure S3C)^28^. In Fourier transform-infrared (FT-IR) spectrum of CQDs, a broad absorption band at about 3300 cm^−1^ was associated with the stretching vibration of N-H and O-H. And the strong peak at about 1690 cm^−1^ was ascribed to the stretching vibration band of C = O^30^. These results confirmed the existence of carboxylic acid and other oxygen-containing functional groups. The peak at about 1515 cm^−1^ corresponded to the stretching vibration and bending vibration bands of N-H, confirming the presence of amino-containing functional groups. Moreover, the absorption bands at 2930, 1410 and 1330 cm^−1^ were assigned to the stretch of vibration C-H, C = C and C-C, indicating the presence of alkyl and aryl groups (Figure S4)^23^. The results were consistent with the XPS analyses. The Transmission electron microscopy (TEM) image revealed that the as-prepared CQDs were well dispersed and possessed a nearly spherical shape (Fig. 4B). The diameter distribution of the CQDs was in the range of 2–8 nm with an average size of about 4.56 nm (Fig. 4B), which was smaller than the CQDs prepared from hydrothermal treatment of hair^23^.

### The response of CQDs to pH values

**Figure 5:**
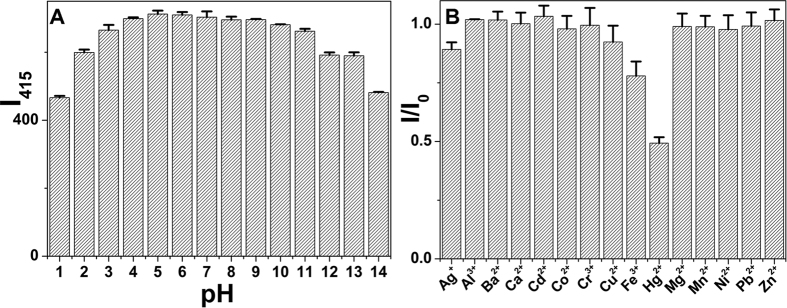
(**A**) Effect of pH on the fluorescence intensity of CQDs. (**B**) Comparison of fluorescence intensities of CQDs in the presence of different ions (Excitation wavelength is 330 nm).

The above results indicated the as-prepared CQDs can be utilized to detect metal ions due to the presence of some functional groups on the surface of CQDs. We first studied the effect of pH on the fluorescence intensity of CQDs in a wide pH range of 1–14. The fluorescence intensity of CQDs was relatively low at a lower pH, indicating the existence of some acidic groups on the surface of CQDs. In contrast, the fluorescence intensity of CQDs remained almost stable in the solution with the pH value of 5–11. And the fluorescence intensity decreased again when the pH value was greater than 11 (Fig. 5A). The CQDs exhibited high stability in aqueous solution with a broad pH range 5–11, indicating that the CQDs have great potential applications in many fields. We chose pH 5.0 for the following experiments.

### The selectivity of CQDs towards metal ions

We further examined the selectivity of CQDs towards different metal ions including Ag^+^, Al^3+^, Ba^2+^, Ca^2+^, Cd^2+^, Co^2+^, Cr^3+^, Cu^2+^, Fe^3+^, Hg^2+^, Mg^2+^, Mn^2+^, Ni^2+^, Pb^2+^ and Zn^2+^. These metal ions at a concentration of 100 μM were added into the CQDs solution, the fluorescence response was recorded. As shown in Fig. 5B, the addition of Hg^2+^ caused a drastic decline in the fluorescence intensity of CQDs, and other metal ions except Ag^+^, Cu^2+^ and Fe^3+^ showed negligible effects, revealing that only Hg^2+^ could strongly interact with CQDs. Ag^+^ is rarely found in tap water. We also found that Cu^2+^ or Fe^3+^ exhibited negligible interference with the detection of Hg^2+^ when these ions were mixed with each other (Figure S5). And triethanolamine can eliminate the interference of Cu^2+^ and Fe^3+^ 31,32. Apparently, the CQDs showed excellent selectivity for Hg^2+^.

### The detection mechanism of CQDs for detecting Hg^2+^

We tried to elucidate the quenching mechanism with the UV-vis absorption spectra and fluorescence lifetime of CQDs. From the UV-vis absorption spectra, we noticed that the addition of Hg^2+^ could cause the disappearance of the two shoulder peaks at about 275 nm and 330 nm (Figure S6), indicating the interaction of CQDs with Hg^2+^. The observation showed the fluorescence quenching mechanism is static quenching process^18^,^33^. The selective interaction of CQDs with Hg^2+^ may originate form the affinity of Hg^2+^ to the carboxyl groups of CQDs^9^,^23^. Meanwhile, the fluorescence lifetime test was also used to elucidate the fluorescence quenching mechanism. Surprisingly, the fluorescence lifetime became longer after the addition of 250 μM Hg^2+^ (5.95 ns) and 500 μM Hg^2+^ (6.22 ns) than that with the CQDs alone (5.15 ns) (Figure S7). The altered fluorescence lifetime signified dynamic quenching^18^,^33^. Accordingly, the fluorescence quenching mechanism of CQDs caused by Hg^2+^ may involve both dynamic and static processes. The response time of CQDs toward Hg^2+^ was also investigated. It was found that the fluorescence of CQDs was immediately quenched by Hg^2+^ within 2 min and remained constant when the incubation time was prolonged to 15 min (Figure S8), allowing the rapid detection of Hg^2+^ without strict time control.

### The sensitivity of CQDs towards Hg^2+^

**Figure 6:**
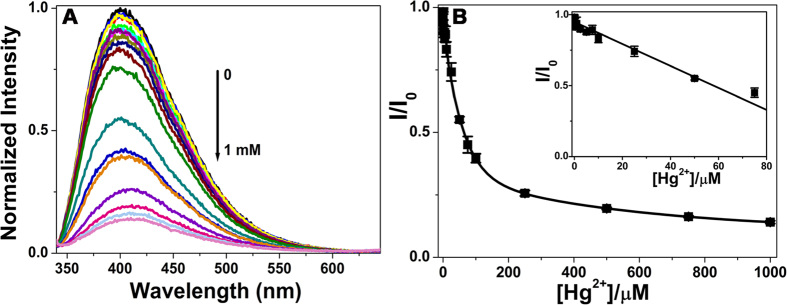
(**A**) Fluorescence emission spectra of CQDs in the presence of different concentrations of Hg^2+^. (**B**) The plot of I/I_0_ versus the concentration of Hg^2+^, the inset is the linear section of the plot (performed in pH 5.0, 50 mM NaAc-HAc buffer; I_0_ and I correspond to the fluorescence intensity of CQDs at 415 nm in the absence and presence of tap water, respectively; excitation wavelength is 330 nm).

We then evaluated the sensitivity of CQDs for Hg^2+^ under the optimized conditions. As shown in Fig. 6A, the fluorescence intensity of CQDs at 415 nm was gradually quenched with increasing the concentration of Hg^2+^ in the range of 0 to 1 mM, the emission peak did not shift obviously. And Fig. 6B showed the plot about the relative fluorescence intensity (I/I_0_) of CQDs versus the concentration of Hg^2+^. Meanwhile, we observed a good linear relationship between I/I_0_ and the Hg^2+^ concentration ranging from 0 to 75 μM, and as low as 0.01 μM Hg^2+^ could be detected. Therefore, the lowest detectable concentration of CQDs for Hg^2+^ was 10 nM, which is comparable with these reported values (Table S1)^9^,^14^,^34^,^35^,^36^,^37^.

### The practical applications of CQDs for detecting Hg^2+^

In order to evaluate the feasibility of CQDs for the detection of Hg^2+^ in real samples, tap water was analyzed in this study. Furthermore, standard addition experiments were performed to detect Hg^2+^. The stepwise addition of Hg^2+^ caused gradual decrease of the fluorescence intensity of CQDs (Figure S9A). The plot of I/I_0_ of CQDs versus the concentration of Hg^2+^ was obtained, and a good linearity between I/I_0_ and the concentration of Hg^2+^ from 0 to 75 μM was obtained, and the correlation coefficient was 0.991 (Figure S9B). These results demonstrated the CQDs had the potential for assaying Hg^2+^ in real water samples.

## Discussion

In the present work, we have demonstrated a facile and low-cost approach for the synthesis of blue-emitting CQDs via thermal treatment of hair. The as-prepared CQDs exhibited excitation-dependent emission behavior, excellent photostability and high stability in high salt conditions. The CQDs can be utilized to detect Hg^2+^ with good selectivity and sensitivity via the Hg^2+^-induced fluorescence quenching mechanism. And the CQDs can detect as low as 10 nM Hg^2+^. The excellent properties and attractive applications make CQDs as promising candidates for monitoring and controlling of environmental pollution.

## Methods

### Materials

Human hair was collected in local barber shop. Hair was thoroughly washed with water and ethanol, and dried in an oven. Other reagents were all of analytical reagent grade and used as received without further purification. The stock solution of different metal ions was prepared with double-distilled water. Double-distilled water was used throughout the experiment.

### Characterization

The UV-vis absorption spectra were recorded at room temperature on a Lambda 650S UV-vis spectrometer (PerkinElmer, USA). The excitation and emission spectra were carried out using a Cary Eclipse luminescence spectrometer (Varian, USA). FT-IR spectra were measured using a Nicolet 5700 FTIR spectrometer (Thermo-Electron Corp., USA) with the KBr pellet technique in the range of 400–4000 cm^−1^. TEM images were performed on a JEM-2100 transmission electron microscope operated at an acceleration voltage of 200 kV. XPS analysis was performed at an X-ray photoelectron spectrometer (ESCALAB 250Xi, Thermo Scientific). Fluorescence lifetime was investigated using a FLS 980 fluorescent spectrofluorimeter (Edinburgh Instrument, UK). The QY of CQDs was calculated using quinine sulphate (in 0.1 M H_2_SO_4_) as a reference. Photostability tests were performed at room temperature using the light of 365 nm from an ultraviolet lamp (16 W, CBIO-UV3A, Beijing CBIO Bioscience Technology Co. Ltd., Beijing, China). Photographs were taken using a Canon 700 D digital camera.

### Synthesis of CQDs

Hair (about 0.2 g) was added into a Teflon-lined autoclave and heated in an oven at a constant temperature of 200 °C for a period of 24 h. After cooling to room temperature, the resulting black solid product was dissolved in water with the assistance of ultrasound. The suspension was centrifuged at 10000 rpm for about 10 min to remove large particles. The brown supernatant was collected for further use. The concentration of CQDs is estimated to be about 16.26 mg/mL from the weight of dried CQDs.

### Detection of Hg^2+^ with CQDs

750 μL acetate buffer solution (NaAc-HAc, 0.2 M, pH 5.0), CQDs solution (30 μL) and different concentrations of Hg^2+^ were mixed together. And water was used to dilute the mixture to final volume of 3 mL (The final concentration of CQDs is about 0.16 mg/mL). After incubation for about 2 min at room temperature, the fluorescence intensity of CQDs was recorded with the fluorescence spectrometer.

### Detection of Hg^2+^ in tap water with CQDs

Tap water was collected in our laboratory and filtered through a 0.22 μm filter. Tap water (60 μL) was mixed with different concentrations of Hg^2+^ and NaAc-HAc buffer. Then CQDs (30 μL) were added into the mixture and water was used to dilute the above mixture to final volume of 3 mL (The final concentration of CQDs is about 0.16 mg/mL). After incubation for about 2 min at room temperature, the fluorescence intensity of CQDs was recorded with the fluorescence spectrometer.

## Additional Information

**How to cite this article**: Guo, Y. *et al*. Thermal treatment of hair for the synthesis of sustainable carbon quantum dots and the applications for sensing Hg^2+^. *Sci. Rep.* **6**, 35795; doi: 10.1038/srep35795 (2016).

## Supplementary Material

Supplementary Information
