# Supplementary material for: Thermal treatment of hair for the synthesis of sustainable carbon quantum dots and the applications for sensing Hg2+
Source: Sci Rep. 2016 Oct 20;6:35795. doi: 10.1038/srep35795 (PMC5071893; doi:10.1038/srep35795)
Supplement: Supplementary Information [file srep35795-s1.pdf]

SUPPORTING INFORMATION

Thermal treatment of hair for the synthesis of sustainable carbon quantum dots and the applications for sensing Hg^{2+}

Yongming Guo[1,*], Lianfeng Zhang[1], Fengpu Cao[1], and Yumin Leng[2,*]

[1]College of Chemistry and Pharmaceutical Engineering, Nanyang Normal University, Nanyang 473061, China

[2]College of Physics and Electronic Engineering, Nanyang Normal University, Nanyang 473061, China

*corresponding authors: Y. G. (email：chinahenangm@163.com) or Y. L. (email: ymleng@nynu.edu.cn).

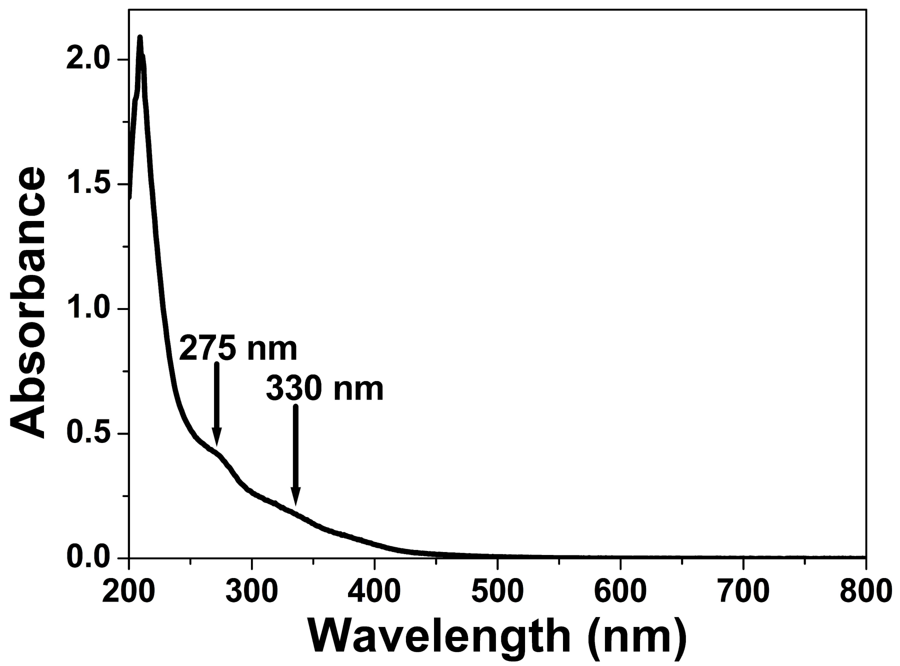

Figure S1 UV-vis absorption spectrum of CQDs.

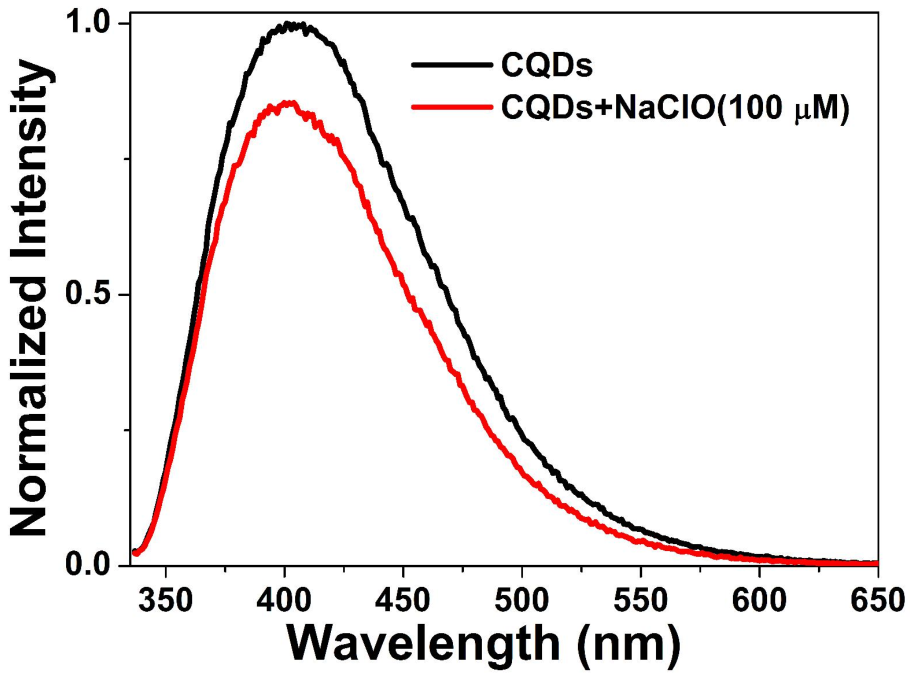

Figure S2 Emission spectra of CQDs in the absence and presence of 100 μM NaClO (Excitation wavelength is 330 nm.).

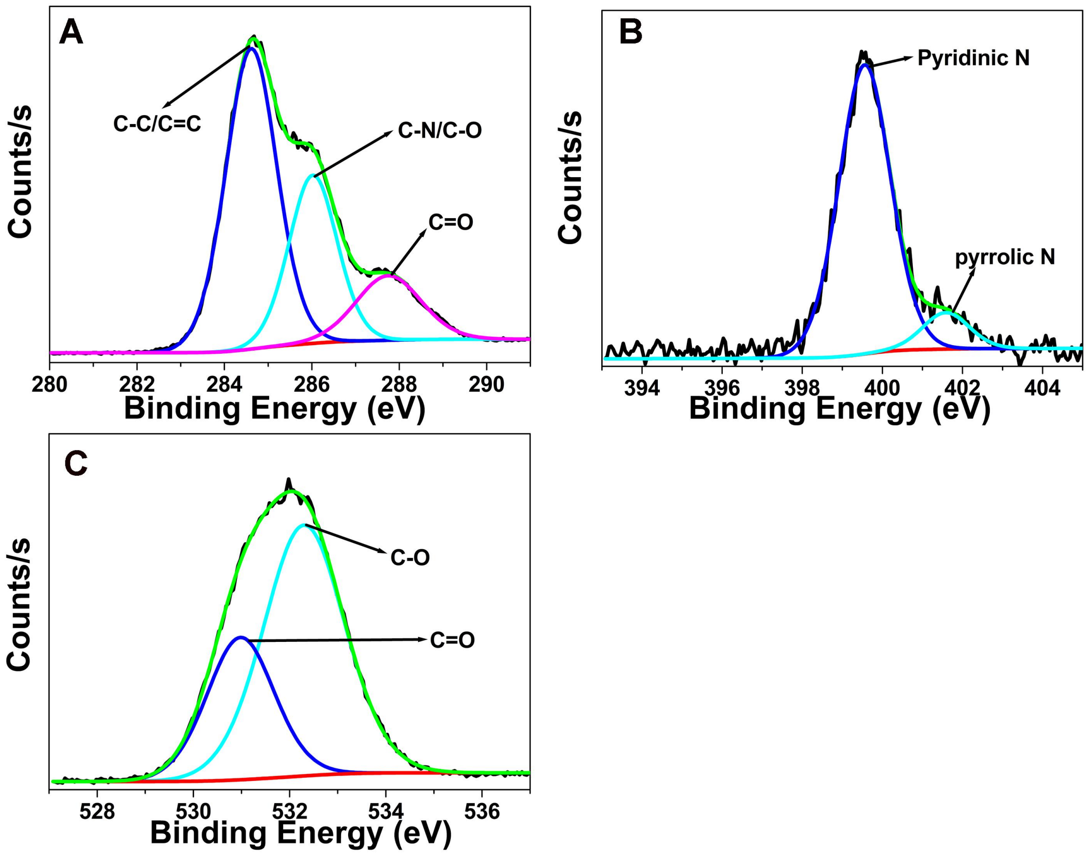

Figure S3 High resolution XPS spectra for C1s, N1s, and O1s of CQDs.

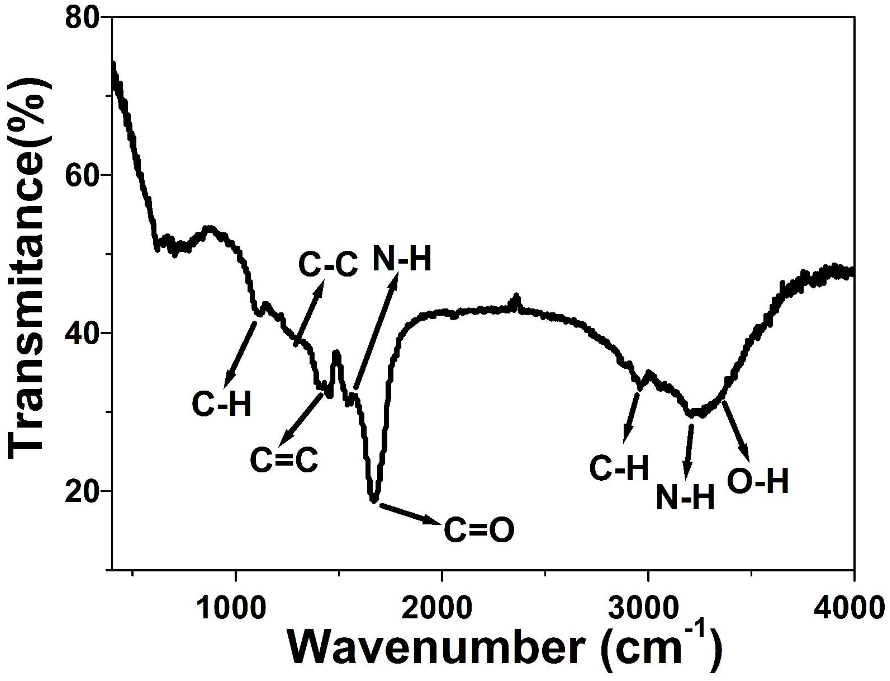

Figure S4 FT-IR absorption spectrum of CQDs.

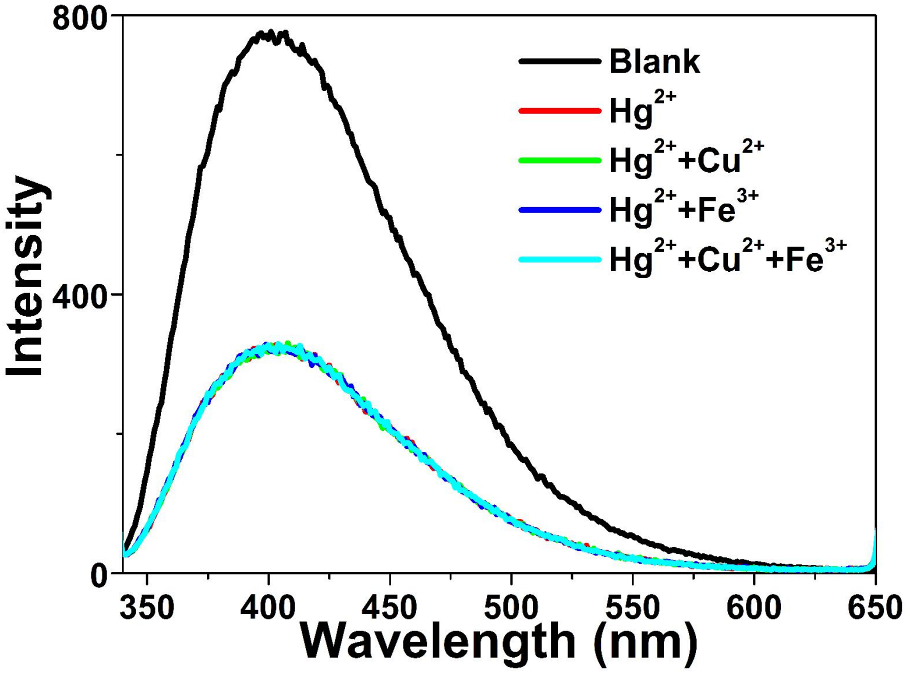

Figure S5 Fluorescence emission spectra of CQDs in the absence and presence of Hg^{2+} (100 μM), Cu^{2+} (20 μM) and Fe^{2+} (20 μM).

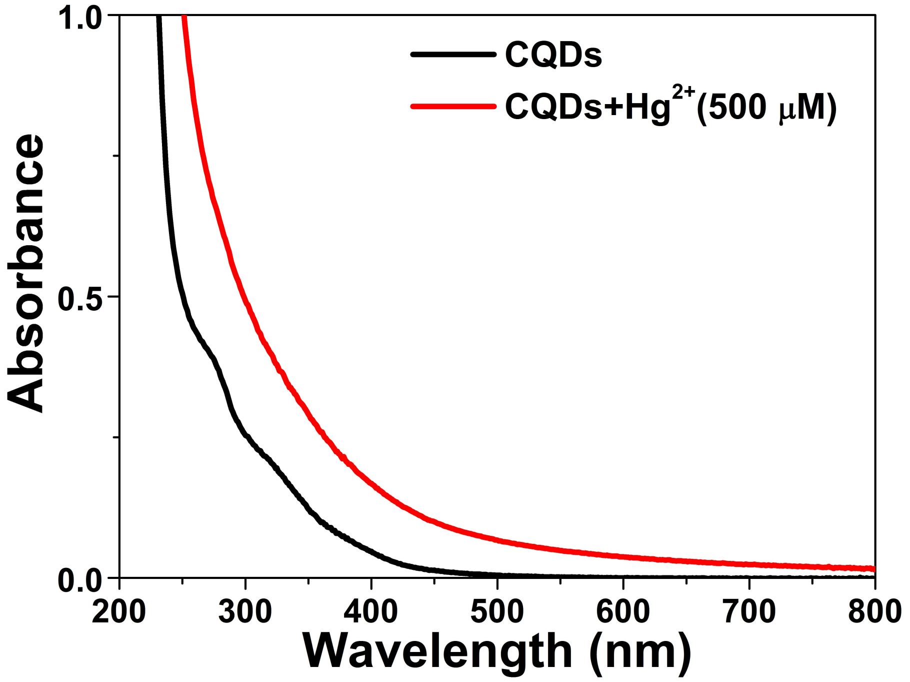

Figure S6 UV-vis absorption spectra of CQDs in the absence and presence of 500 μM Hg^{2+}.

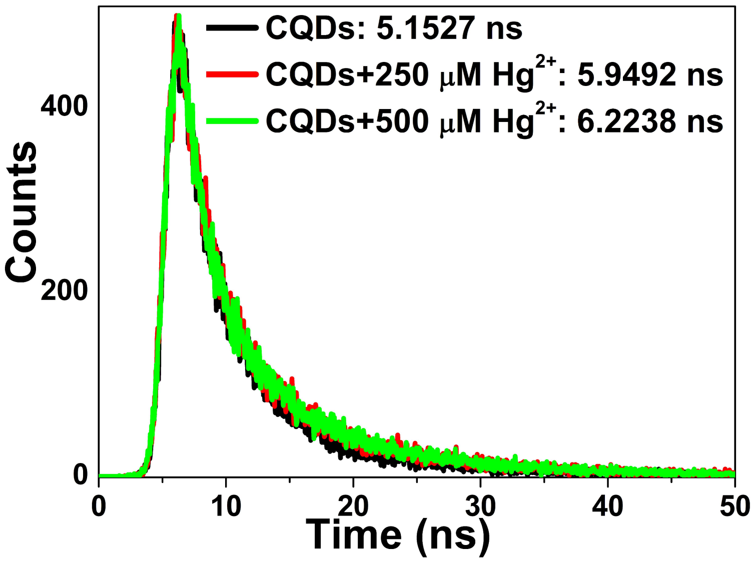

Figure S7 Fluorescence decay curve of CQDs in the absence and presence of 250 μM and 500 μM Hg^{2+} (Excitation wavelength is 330 nm, emission wavelength is 415 nm.).

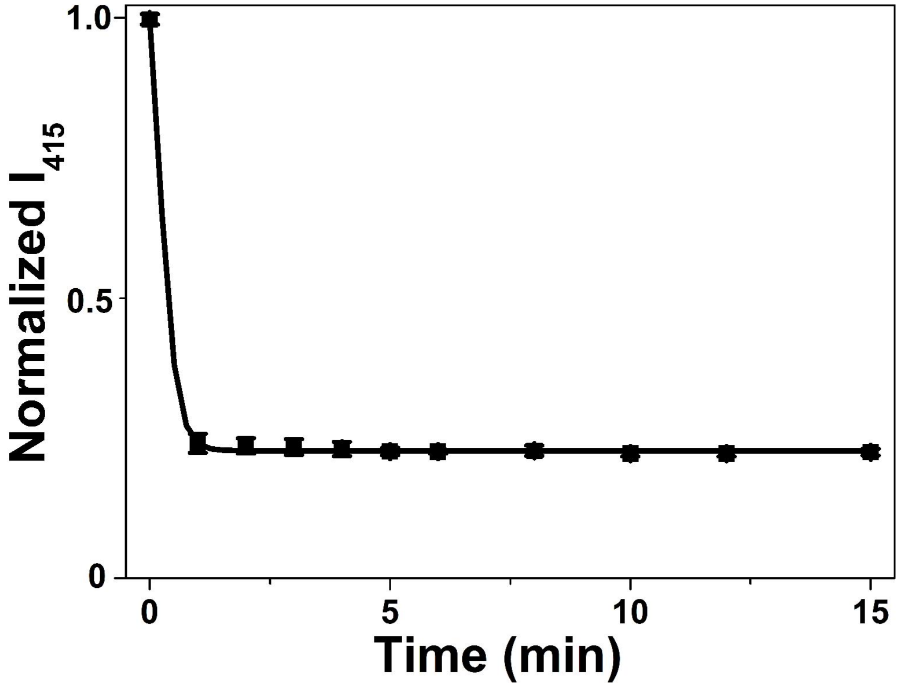

Figure S8 The effect of incubation time on the fluorescence intensity of CQDs at 415 nm in the presence of 500 μM Hg^{2+} (performed in pH 5.0, 50 mM NaAc-HAc buffer; excitation wavelength is 330 nm.).

Table S1. Comparison of the performance of different CQDs for Hg^{2+} detection.

Probes	Detection limit	Linear range	Quantum yield (%)	Reference
CQDs	10 nM	0-5 μM	68	1
Nitrogen-doped CQDs	0.23 μM	0-25 μM	15.7	2
Carbon nitride quantum dots	0.14 μM	0.1-10 μM, 10-30 μM	27.1	3
Eu^{3+}/CQDs@MOF-253	13 nM	0-150 μM	42.3	4
CQDs	1.6 μM	6-80 μM	4.7	5
CQDs	1.51 nM, 126 nM	0-10 nM, 100-5000 nM	44.8	6
CQDs	10 nM	0-75 μM	10.75	This work

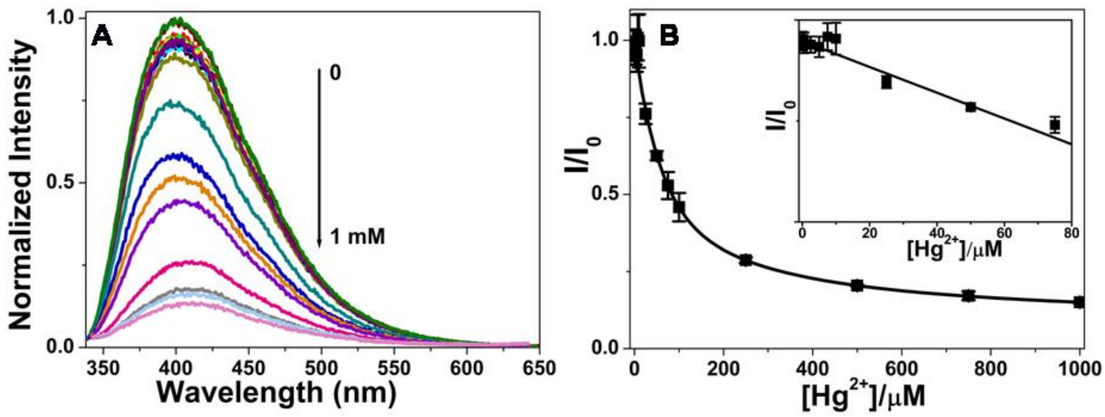

Figure S9 (A) Fluorescence emission spectra of CQDs in the presence of different concentrations of Hg^{2+} in tap water. (B) The plot of I/I_0 versus the concentration of Hg^{2+} in tap water, the inset is the linear section of the plot (performed in pH 5.0, 50 mM NaAc-HAc buffer; I_0 and I correspond to the fluorescence intensity of CQDs at 415 nm in the absence and presence of Hg^{2+}, respectively; excitation wavelength is 330 nm.).

References:

1. Guo, Y., Wang, Z., Shao, H., & Jiang, X., Hydrothermal synthesis of highly fluorescent carbon nanoparticles from sodium citrate and their use for the detection

of mercury ions. *Carbon* **52**, 583-589 (2013).

2. Zhang, R. & Wei, C., Nitrogen-doped carbon quantum dots: Facile synthesis and application as a "turn-of" fluorescent probe for detection of Hg^{2+} ions. *Biosens. Bioelectron.* **55**, 83-90 (2013).
3. Cao, X. *et al.*, A facile microwave-assisted fabrication of fluorescent carbon nitride quantum dots and their application in the detection of mercury ions. *Spectrochim. Acta A.* **151**, 875-880 (2015).
4. Xu, X.-Y. & Yan, B., Fabrication and application of a ratiometric and colorimetric fluorescent probe for Hg^{2+} based on dual-emissive metal-organic framework hybrids with carbon dots and Eu^{3+}. *J. Mater. Chem. C* **4**, 1543-1549 (2016).
5. Wang, C. *et al.*, Synthesis of cellulose-derived carbon dots using acidic ionic liquid as a catalyst and its application for detection of Hg^{2+}. *J. Mater. Sci.***51**, 861-867 (2015).
6. Zhang, C., Hu, Z., Song, L., Cui, Y., & Liu, X., Valine-derived carbon dots with colour-tunable fluorescence for the detection of Hg^{2+} with high sensitivity and selectivity. *New J. Chem.* **39**, 6201-6206 (2015).
